# “Does one plus one exceed two?” The synergistic effect of Innovative City and Smart City pilots on work safety governance—evidence from a quasi-natural experiment

**DOI:** 10.3389/fpubh.2025.1720609

**Published:** 2025-12-19

**Authors:** Liqing Li, Peisong Han, Mengjie Xu

**Affiliations:** 1 College of Public Administration and Law, Hunan Agricultural University, Changsha, Hunan, China; 2 College of Sociology, Guizhou Minzu University, Guiyang, Guizhou, China

**Keywords:** Innovative City, Smart City, work safety governance, dual pilot policy, synergistic effect

## Abstract

Work-safety governance is a fundamental pillar for aligning high-quality development with robust safety standards and serves as a critical safeguard for workers’ rights to life and decent work. Utilizing panel data from 218 Chinese cities spanning 2008–2023 and employing a staggered difference-in-differences (DID) model, this study investigates whether the dual pilot programs for Innovative Cities and Smart Cities generate a synergistic effect, where “1 + 1 > 2,” that enhances urban work-safety governance outcomes. The key findings are as follows: (i) The dual-pilot policy significantly enhances work-safety governance through synergy. Compared to non-pilot and single-pilot cities, dual-pilot cities experience an average reduction of 69.8 and 38.8 fatalities in work-safety accidents, respectively. Moreover, the “innovation-first” implementation sequence yields a notably stronger synergy than the “smart-first” pathway. (ii) Mechanism analyses reveal that the policy improves work-safety governance primarily via two channels: technological innovation (TI) and industrial upgrading (IU), with safety regulation (SR) positively moderating the policy’s effectiveness during implementation. (iii) The spatial spillover effect of the dual-pilot policy follows a distance-decay pattern characterized by promotion in nearby areas, suppression in mid-to-far areas, and disappearance in distant areas. Specifically, the policy promotes safety governance within 800 km, exerts suppressive effects between 800 and 1,400 km, and its influence essentially vanishes beyond 1,400 km. (iv) Heterogeneity tests demonstrate that the dual-pilot policy’s governance effects are more pronounced in non-resource-based cities, small- and medium-sized cities, and cities located in the central region. This study contributes new empirical evidence on the synergistic mechanisms of urban safety governance within a risk society context and provides valuable policy insights for constructing more efficient urban work-safety governance systems.

## Introduction

**Figure 1:**
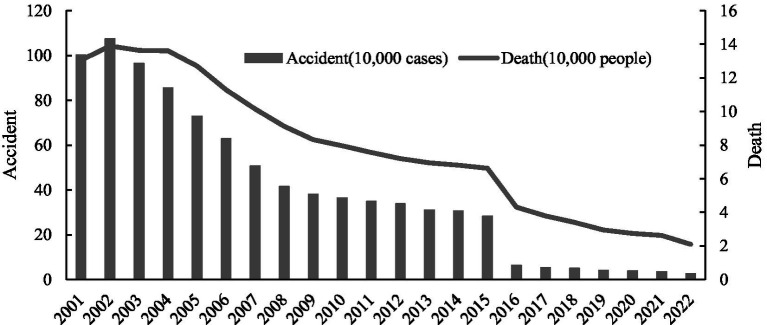
Trends in work safety accident and death in China.

Safety is a fundamental prerequisite for human production and development. Amid rapid urbanization and industrialization, various systemic risks have continued to accumulate, prompting the Chinese government to elevate urban safety and resilience building to the level of a national strategy aimed at effectively safeguarding people’s lives and property. This strategic emphasis is reflected in a series of policy milestones: from the 2015 Central Urban Work Conference’s explicit call to “put safety first,” to the 2018 Opinions on Promoting Urban Safety Development, which proposed constructing an urban safety assurance system focused on preventing and curbing particularly serious work-safety accidents, and then to the 2025 new round of the Central Urban Work Conference, which advocates building a modern, people-centered city that is “innovative, livable, beautiful, resilient, civilized, and smart.” Together, these policy developments illustrate China’s sustained commitment to deepening and strategically upgrading the coordination of urban safety and development. Work-safety accidents represent a prominent risk in urban operations due to their high frequency, wide scope, and severe casualties. The governance outcomes of such accidents directly impact the quality of the overall urban safety framework. As shown in Figure 1, from 2001 to 2022, China reported approximately 8.8 million work-safety accidents, resulting in about 1.7 million deaths. Although the number of accidents and fatalities has significantly declined over the past decade, the current work-safety situation remains severe. This stark reality underscores the urgency of effective risk governance in work safety and highlights that improving work-safety governance effectiveness is a vital foundation for achieving the United Nations sustainable development goals (SDGs), particularly SDG 3 (Good Health and Well-being) and SDG 8 (Decent Work and Economic Growth).

To enhance work-safety governance effectiveness, the Chinese government has actively promoted the coordinated deployment of multiple policy instruments. Among these, the Innovative City pilot and the Smart City pilot, hereafter referred to as the “dual-pilot policy,” stand out as representative initiatives offering governance support through systemic innovation and smart empowerment, respectively. The Innovative City pilot emphasizes technological innovation (TI), industrial transformation, and institutional optimization (1), thereby establishing a systemic safeguard for work safety. Meanwhile, the Smart City pilot leverages new-generation information technologies such as the internet of things (IoT), big data, and artificial intelligence (AI) (2), to advance urban governance toward intelligence and refinement. By developing urban operation sensing platforms, intelligent monitoring and early-warning systems, and collaborative emergency response mechanisms, it enables real-time monitoring, dynamic risk assessment, and efficient management of safety hazards (3, 4). This technology-empowered governance pathway aligns closely with advanced international frameworks. For example, the European agency for safety and health at work (EU-OSHA) advocates fully utilizing digital technologies like IoT and AI to prevent occupational safety risks and reduce work-related accidents and diseases (5). Similarly, the OECD report Smart Cities and Inclusive Growth highlights smart city development as a means to enhance social well-being, safety, and sustainability (6). The organic integration of innovation-driven and smart empowerment enables these two pilots to achieve complementary advantages, overcoming the limitations of single policies and jointly supporting a precise, coordinated, and modern urban public safety governance system. This study empirically examines the synergistic effects and mechanisms through which the dual-pilot policy influences work-safety governance in Chinese cities.

Scholars have extensively studied work-safety governance measures and their impacts. At the enterprise level, efforts include increased safety investments, standardized operational behaviors, construction of industrial chain safety governance networks for knowledge and information sharing (7), improving conditions for small and medium-sized enterprises (SMEs) (8), and encouraging broader work-safety commitments among companies (9). At the government level, numerous studies emphasize the role of scientific and reasonable regulation in safeguarding workers. Jiang et al. argue that integrating work-safety performance into officials’ promotion criteria, with “one-vote veto” power, incentivizes local governments to prioritize worker safety, effectively reducing work-related accidents driven by economic development (10). Fisman et al. further demonstrate that this “no safety, no promotion” mechanism lowers fatality rates in politically connected enterprises by 86%, compared to a 30% reduction in others (11). Central government measures, including mandatory oversight (12), information disclosure (13), administrative accountability (14), and the national civilized city campaign (15) exert political pressure on local governments to improve occupational safety, encouraging enterprises to enhance their safety environments, thus reducing worker injuries and fatalities and promoting decent labor. Research also links officials’ personal characteristics, tenure changes, and corruption to accident rates and fatalities (10, 12, 16, 17). Slil et al. contend that strong safety leadership enhances organizational risk response and fosters employee safety responsibility awareness, encouraging collective action against occupational risks (18).

Nonetheless, systemic defects in traditional safety governance remain a root cause of work-related accidents (19). The advent of Industry 4.0 has fundamentally reshaped work-safety governance, profoundly affecting occupational health and safety management (20). In this context, the roles of Innovative and Smart City development in enhancing governance are increasingly prominent. Research indicates that Innovative City development accelerates technological progress, industrial upgrading (IU), and institutional innovation, thus providing systemic support for work safety (21–23). Conversely, Smart City initiatives use new-generation information technologies to enable real-time monitoring, intelligent early warning, rapid response, and precise governance of urban safety risks, significantly improving work-safety outcomes (3) However, given complex and evolving production environments and hidden risks, single policies often fall short of desired governance objectives (24). Coordinated policy implementation facilitates alignment and complementarity, creating a more comprehensive system that generates synergistic efficiency gains. Accordingly, scholars have shifted focus from single-policy impacts to the synergistic effects of dual or multiple pilot policies. For instance, coordinated implementation of Smart City and Innovative City pilots has shown greater effectiveness than single policies in promoting digital economy development (25), nurturing new productive forces (26), and advancing green, low-carbon development (27).

Despite valuable insights, existing research has limitations. First, most studies focus on single-policy impacts on work-safety governance, lacking exploration of synergistic effects from multiple policies, potentially biasing governance outcome estimates. In particular, research on the collaborative governance effects of Innovative City and Smart City pilots remains limited. Second, while acknowledging spatial spillover effects of policy implementation, existing literature rarely measures the geographic decay boundaries of these spillovers, limiting understanding of the spatial reach of policy impacts. Addressing these gaps, this study adopts a policy synergy perspective to systematically analyze the impacts, mechanisms, and spatial spillover boundaries of the Innovative City and Smart City pilots on work-safety governance. The goal is to provide theoretical and empirical insights for building a more inclusive, sustainable, and resilient urban safety governance system.

This paper’s potential contributions include: (i) From a research standpoint, integrating innovation-driven and smart empowerment policy logics provides a novel analytical lens to understand how combined policies synergistically enhance work-safety governance effectiveness, expanding the public value of work safety to encompass workers’ safety, health, and decent work; (ii) In terms of identification strategy, using quasi-natural experiment-induced inter-city pilot timing differences and a multi-period difference-in-differences (DID) model allows for precise estimation of the dual-pilot policy’s “net effect” on governance outcomes by comparing pilot and non-pilot cities over time; (iii) By conducting rolling regressions at 100 km intervals, this study offers a detailed measure of geographic decay boundaries, systematically characterizing spatial spillovers, including their driving distance, siphoning zones, and attenuation thresholds, thereby providing robust evidence on the externalities and spatial limits of pilot policies.

## Policy context

The Innovative City pilot policy is a cornerstone of China’s innovation-driven development strategy and a key driver in transforming economic growth models. Since its inception in Shenzhen in 2008, the policy has been expanded through seven rounds of pilot projects. By 2025, a total of 97 prefecture level cities, approximately 29.13% of all cities nationwide, have been included in the pilot program. A review of policy documents reveals three defining characteristics of the Innovative City pilot policy. First, it fosters collaboration between government and market forces. Local governments coordinate the overall advancement of the initiative, while the market plays a decisive role in allocating innovation resources, creating a dynamic synergy between proactive governance and effective market mechanisms. Second, the policy aims to establish an efficient innovation production system by attracting innovative talent, increasing financial investment, strengthening protection of intellectual property, and providing comprehensive innovation platforms. These efforts continually optimize the urban innovation environment, promote the clustering of high-end industries, and comprehensively enhance cities’ innovation capacity and overall competitiveness. Third, there is a strong emphasis on transforming innovative achievements into high-quality development. The policy seeks to lead in key technologies to accelerate the formation of globally competitive modern industrial systems and to promote industrial structure optimization and upgrading. Simultaneously, “safety and environmental protection” are fundamental principles, with technological progress driving the economy and society toward safe, green, low-carbon, and sustainable development.

The Smart City pilot policy integrates new-generation information technologies, including the IoT, cloud computing, big data, and AI, into the full spectrum of urban planning, construction, management, and services. This drives the transformation of urban governance toward greater intelligence, refinement, and modernization. The policy was officially launched in 2012 when China’s Ministry of Housing and Urban-Rural Development issued the Notice on Carrying Out National Smart City Pilot Work and announced the first batch of pilot cities. Since then, China has implemented three rounds of Smart City pilots, encompassing 104 prefecture level cities, or roughly 31.23% of the country’s total cities. In August 2025, the Central Committee of the Communist Party of China and the State Council issued the Opinions on Promoting High-Quality Urban Development, further underscoring Smart City construction as a core strategy to enhance urban governance effectiveness and overall capacity, with the ultimate goal of improving urban residents’ sense of gain, happiness, and security.

## Theoretical analysis and research hypotheses

### Direct impact of combining innovation and smart-city development on work safety governance

Innovative City and Smart City represent two complementary pathways for achieving high-quality development and elevated safety standards in Chinese cities. Together, they form key pillars of modern urban governance by reinforcing each other’s objectives and mechanisms. An Innovative City focuses on enhancing indigenous innovation, optimizing the innovation environment, aggregating innovation resources, cultivating innovation actors, and renewing development concepts. This fosters industrial transformation and upgrading, sustains momentum for growth, and provides crucial technological and institutional support for urban safety governance (23, 28). Conversely, a Smart City applies technologies such as the IoT, big data, AI, blockchain, and cloud computing to intelligentize infrastructure, public services, social governance, and the ecological environment. This shift enables a move from passive responses to proactive intelligent early warnings, significantly boosting capabilities in risk perception, integrated assessment, rapid response, and precise control (4, 29).

These two development modes interact closely. Innovative Cities drive continuous technological breakthroughs and institutional innovations, for example, advanced sensing devices, intelligent decision algorithms, and cross-departmental coordination mechanisms. Meanwhile, Smart Cities construct city-wide perception systems and dynamic information models that provide real-world application scenarios and validation environments for innovative technologies, thereby facilitating technology iteration and policy optimization (30). Furthermore, the complex systemic challenges, emerging risks, and demand for advanced intelligent technologies revealed through smart governance continuously stimulate innovation entities to engage in research and development, creating a reinforcing closed loop of “innovation-driven, smart enablement” (31, 32). This synergistic mechanism significantly enhances work-safety governance: Innovative Cities reduce accident risks at the source through process improvements, safety technology R&D, and management reforms; Smart Cities employ intelligent sensors, risk-simulation models, and emergency-command platforms to achieve comprehensive risk perception, intelligent assessment, and rapid response to safety threats, thereby minimizing losses (33, 34). Based on this, we propose Hypothesis 1.

*H1*: Dual pilot city construction significantly improves work safety governance outcomes.

#### Mechanisms by which the combined model affects work safety governance

##### Technological-innovation effect

TI is a primary driver of improved work-safety governance. First, as a vital source of cutting-edge safety technologies, Innovative Cities employ subsidies, tax incentives, talent programs, funding, and industry–university–research platforms to encourage mission-oriented R&D by firms and research institutes (35), resulting in continuous advancements in practical safety technologies. Notably, breakthroughs in safety equipment and robotics have led to high-performance protective gear, firefighting robots, and hazardous-materials-disposal robots, significantly enhancing the substitution of human operations in extreme environments and improving disaster-response capabilities (36–38). Innovations in automation and intelligent processes enable unmanned operations at high-risk posts, thereby reducing error risks associated with manual intervention at the source (39). Additionally, advances in intelligent perception and predictive technologies, supported by high-precision sensors and machine vision, allow intelligent monitoring systems to detect violations, equipment leaks, and other hazards in real time (38); When combined with big-data models analyzing multi-source data, these systems facilitate accurate risk forecasting and efficient emergency response (40). Second, Smart Cities, through IoT perception networks, data-algorithm centers, and intelligent emergency platforms, provide an application environment for these advanced technologies, promoting system-level integration and utilization (41), Real-world feedback further drives ongoing optimization (42). This synergy between Innovative and Smart Cities reduces risk exposure in hazardous environments, enhances sensitivity in risk identification, and optimizes scientific decision-making and emergency response procedures, thereby comprehensively improving work-safety governance. Based on this, we propose Hypothesis 2.

*H2*: Dual pilot city construction improves work safety governance by promoting technological progress.

##### Industrial-upgrading effect

Industrial structural transformation and upgrading is a crucial pathway for reducing work-safety accidents. In the coordinated development of Innovative Cities and Smart Cities, the transformation or phasing out of high-risk industries alongside the cultivation of low-risk emerging sectors can significantly lower the incidence of work-related accidents. First, driven by policy guidance and TI, local governments progressively renovate, relocate, or eliminate industries characterized by pronounced safety risks, outdated technologies, and extensive management deficiencies. This process reduces urban dependence on the second industry, where work-safety accidents are most frequent, thereby controlling risks at the source and elevating overall safety levels. Second, local governments actively promote the growth of low-risk emerging industries, such as high-tech sectors, intelligent manufacturing, and modern services, facilitating a shift toward knowledge- and technology-intensive economic activities (43, 44). Third, as national emphasis on safe development increases, the integration of the two city programs further nurtures safety-related industries. This fosters the gradual formation of a comprehensive industrial chain covering safety equipment production, maintenance, equipment servicing, safety tool R&D, software updates, operations management, safety training, and testing and certification, providing system-wide support for work-safety governance. In sum, the synergy of Innovative and Smart City models drives the industrial system toward higher-end, greener, and safer development. It strengthens the work-safety governance framework and reduces accident incidence through industrial structure optimization, thereby improving overall governance outcomes. Based on this, we propose Hypothesis 3:

*H3*: Dual pilot city construction improves work safety governance by optimizing the industrial structure.

##### Safety-regulation effect

Safety regulation (SR) refers to the mandatory measures implemented by the government to ensure that enterprises fulfill their primary responsibility for work safety. Typically, governments employ administrative licenses, technical standards, educational training, law enforcement inspections, and other methods to regulate business operations (45, 46), prevent safety accidents, and enhance the effectiveness of work-safety governance (47). Both SR and the dual-pilot policies represent crucial institutional arrangements in China aimed at preventing work-safety accidents. According to institutional complementarity theory, these two systems are not isolated but rather form a mutually supportive and synergistic institutional framework. First, SR exerts a normative and constraining influence on TI and its application. During the simultaneous development of Innovative and Smart Cities, both governments and enterprises may tend to allocate resources toward areas with immediate economic returns, potentially neglecting research and development in safety technologies. SR establishes clear technical standards and legal responsibilities, setting a baseline for the development and deployment of new production technologies. This ensures that TI advances economic growth while maintaining safety considerations. Without regulatory oversight, even the most advanced technologies may introduce new risks due to misuse or misapplication (48, 49). Second, SR acts as a catalyst for the adoption and promotion of new technologies. Strict laws and enforcement encourage enterprises to proactively introduce innovative technologies and practices to meet compliance requirements, thereby enhancing safety management, improving production processes, and standardizing operational behaviors. This process reinforces the positive effects of the dual-pilot policies on work-safety governance. Simultaneously, a clear and stable regulatory environment reduces policy uncertainty for enterprises adopting new technologies, bolsters investment confidence, and provides legitimacy and institutional support for technology promotion. These factors reduce transaction costs and facilitate the diffusion and penetration of safety innovations into governance practices. Additionally, from the perspective of full-process governance, SR systematically enhances work-safety governance effectiveness through measures such as pre-production safety education, in-process hazard identification, and post-production punishment and accountability (50). Based on these insights, the following hypotheses are proposed:

*H4a*: SR plays a positive moderating role in the process of the dual-pilot policies enhancing work-safety governance effectiveness.

*H4b*: The interaction between SR and the dual-pilot policies produces governance effects that exceed their individual effects.

## Research design

### Model construction

By 2023, China had launched seven batches of Innovative City pilots (in 2008, 2010, 2011, 2012, 2013, 2018, and 2022), covering 97 prefecturelevel cities. Additionally, three batches of Smart City pilots were introduced in January 2013, August 2013, and April 2015, encompassing 104 prefecture level cities. Due to the differences in city lists and implementation timings, this study employs a staggered DID approach to estimate the impact of the dual-pilot policy on work-safety governance.

The baseline model is denoted by Equation 1.


$$\text{Safet}y_{\mathrm{it}}=\alpha_{0}+\alpha_{1}\text{Dual}\_\text{polic}y_{\mathrm{it}}+\alpha_{2}X_{\mathrm{it}}+\mu_{i}+t_{i}+\varepsilon_{\mathrm{it}}$$ (1)

In Equation 1, *i* indexes cities and *t* indexes years. The dependent variable, Safetyit represents the work-safety governance outcome for city *i* in year *t*. The core explanatory variable is *Dual_policy_it_*. Control variables are also included, along with a constant term. The model estimates the parameters of the core explanatory and control variables while incorporating fixed effects for both city and year. The error term is specified as a random disturbance.

To explore the underlying mechanisms, we test mediation effects through technological progress and industrial upgrading, as well as the moderating effect of SR. The mediation models are represented by Equations 2 and 3.


$$M_{\mathrm{it}}=\beta_{0}+\beta_{1}\text{Dual}\_\text{polic}y_{\mathrm{it}}+\beta_{2}X_{\mathrm{it}}+\mu_{i}+t_{i}+\varepsilon_{\mathrm{it}}$$ (2)

$$\text{Safet}y_{\mathrm{it}}=\beta_{0}+\beta_{1}\text{Dual}\_\text{polic}y_{\mathrm{it}}+\beta_{2}M_{\mathrm{it}}+\beta_{3}X_{\mathrm{it}}+\mu_{i}+t_{i}+\varepsilon_{\mathrm{it}}$$ (3)

In Equations 2 and 3, the mediator is denoted as Mit; and a constant term *β*_0_ is included. The coefficients *β*₁, *β*₂, and *β*₃ are estimated, while other variables remain consistent with those in Equation 1.

For the moderation analysis, we incorporate SR and its interaction with the dual-pilot policy, resulting in Model 4.


$$\begin{array}{l} \text{Safet}y_{\mathrm{it}}=\rho_{0}+\rho_{1}\text{Dual}\_\text{polic}y_{\mathrm{it}}+\rho_{2}SR_{\mathrm{it}}+\rho_{3}\text{Dual}\_\text{polic}y_{\mathrm{it}}\\ \times R_{\mathrm{it}}+\rho_{4}X_{\mathrm{it}}+\mu_{i}+t_{i}+\varepsilon_{\mathrm{it}} \end{array}$$ (4)

In Equation 4, the moderator is SR, denoted as SR*_it_*, and the interaction term is the product of the dual-pilot policy and SR, expressed as *Dual_policy_it_* × SR*_it_*; A constant term *ρ*₀ is included, and the coefficients *ρ₁*, *ρ₂*, *ρ₃*, and ρ₄ are estimated. Other variables remain consistent with those in Equation 1.

### Variable selection

#### Dependent variable

We use work-safety governance as the dependent variable. Following Zuo et al. (49) and Li et al. (50), we measure it inversely by the number of deaths from work-safety accidents, fewer deaths indicate better governance. To further verify robustness, this study also employs the death rate per 100 million yuan of GDP as an alternative indicator.

#### Explanatory variables

The dual-pilot policy serves as the core explanatory variable in this study and is represented by the dummy variable Dual_policy, constructed as the interaction of Policy and Post. The Policy variable identifies whether a city i is part of the pilot program: a city simultaneously designated as both an “Innovative City” and a “Smart City” pilot is classified as the experimental group and assigned a value of 1; otherwise, it belongs to the control group and is assigned 0. The Post variable distinguishes the periods before and after policy implementation. For cities in the experimental group, Post is 0 prior to the implementation year of the dual-pilot policy and 1 in the year of implementation and onwards. The “single-pilot” policy variable and the dual-pilot variable for subsequent analyses are constructed using the same approach.

#### Mechanism variables

##### TI

TI plays a crucial role in enhancing work-safety governance by reducing human-error risks and enabling unmanned operations at high-risk posts. Under the dual-pilot framework, governments increase innovation inputs and foster factor agglomeration to drive breakthroughs in safety technologies. Through transformation platforms, these innovations are promoted for practical application in safety management. We proxy technological progress using the number of patent applications, as this indicator captures the entire innovation process from research and development to practical transformation.

##### Industrial upgrading

The secondary sector, particularly manufacturing, has historically been a high-risk domain for work-safety accidents, including industries such as mining, metallurgy, non-ferrous metals, building materials, machinery, light industry, textiles, tobacco, commerce, hazardous chemicals, and fireworks. The dual-pilot policy offers an opportunity to upgrade the secondary sector by promoting TI and digital transformation. This policy encourages traditional manufacturing to evolve toward high-end, intelligent development. The adoption of smart manufacturing and automation optimizes production processes, increases efficiency, reduces manual operations, and enhances early warning systems, collectively lowering the probability of accidents. Industrial upgrading is proxied by the share of secondary-industry value added in GDP.

##### SR

We use “Safety Attention” as a proxy for regulatory strength. According to attention-allocation theory, government attention is a scarce administrative resource, and its allocation reflects policy priority and regulatory intensity. Specifically, (i) we construct a keyword list related to work safety, hidden-hazard inspections, special rectifications, and similar topics; (ii) through text analysis, we systematically extract the frequency of these safety-related terms from municipal government work reports spanning 2008–2023; and (iii) we calculate the proportion of safety-related word counts relative to the total word count to quantify local regulatory intensity.

#### Control variables

Drawing on the studies by Jiang and Chai (10), Wang and Wang (12), Zhou et al. (16), and Li et al. (50), the following control variables are selected: economic development level (lnGDP), public fiscal expenditure (PF), technological research and development investment (TR), fixed asset investment (FA), urbanization level (UL), and industrial employment rate (ER).

### Data sources

This study uses data from Chinese prefecture-level cities spanning 2008 to 2023 for empirical analysis. The choice to focus on prefecture-level cities, rather than provincial-level administrative regions, is based on three main reasons: First, the implementation and management of the Innovative City and Smart City pilot policies are carried out at the prefecture-level city government. Policy execution and resource allocation occur primarily at the city level. Using provincial-level data would aggregate pilot and non-pilot cities within the same province, making it difficult to accurately identify the true effects of the dual-pilot policies and potentially causing estimation bias. Second, significant differences exist among prefecture-level cities in terms of industrial structure, risk types, and government governance capacity. These differences result in heterogeneous impacts of the dual-pilot policies on work-safety governance effectiveness. Prefecture-level data allows for the identification of differentiated policy effects across diverse city types, thereby addressing the question of which cities benefit most from the policy. Finally, the prefecture-level city sample offers a larger number of observations and greater variability, enhancing the degrees of freedom for statistical inference and improving the robustness of model estimates.

After excluding cities with administrative boundary changes or severe data missingness, the final sample comprises 218 cities and 3,488 observations. Data on accident deaths and deaths per 100 million yuan of GDP are sourced from Statistical Communiqués in local national economic and social development reports. SR data is derived from municipal government work reports. Data on technology, industry, and control variables come from the China City Statistical Yearbook and local yearbooks, with limited interpolation applied to address missing values. Descriptive statistics are presented in Table 1.

**Table 1:** Descriptive statistics.

Variable	Definition	Mean	Std. dev.	Min	Max
Safety	Death toll from work safety accidents (in hundreds)	1.397	1.455	0.000	13.490
Dual_policy	=1 for the year when a city began participating in both types of pilots and all years after; otherwise 0	0.085	0.279	0.000	1.000
TI	Logarithm of patent application data	8.234	1.571	3.258	12.599
IU	Proportion of the secondary industry’s value-added to GDP (%)	0.462	0.111	0.107	0.910
SR	Proportion of safety-related keyword frequency to total word frequency in government work reports (%)	0.002	0.002	0.000	0.017
lnGDP	lnGDP	3.179	0.424	1.880	4.539
PF	Proportion of public fiscal expenditure to GDP (%)	0.193	0.105	0.044	1.027
TR	Proportion of technological expenditure to public fiscal expenditure (%)	0.018	0.018	0.001	0.207
FA	Proportion of fixed asset investment to GDP (%)	1.029	0.782	0.026	12.446
UL	Population size (ten thousand people)	438.204	266.681	18.590	1604.110
ER	Proportion of employment in the secondary industry (%)	43.819	13.827	4.460	84.400

## Empirical results

### Baseline regression results

Table 2 presents the baseline estimates of the dual pilot’s effect on work safety governance. Column (1) shows the estimates without control variables, while column (2) includes control variables. In both specifications, the coefficient on Dual_policy is negative and statistically significant at the 1% level, indicating a significant improvement in work-safety governance and thereby confirming Hypothesis 1.

**Table 2:** Baseline regression results.

Variables	(1)	(2)	(3)	(4)	(5)	(6)
Dual_policy	−0.227**	−0.555***			−0.698***	−0.388***
	(0.088)	(0.075)			(0.074)	(0.092)
Innova_policy			−0.576***			
			(0.081)			
Smart_policy				−0.376***		
				(0.068)		
lnGDP		−0.042	0.493*	0.565**	−1.097***	−0.055
		(0.261)	(0.260)	(0.262)	(0.302)	(0.435)
PF		−0.094	−0.168	0.125	−0.992*	−0.117
		(0.431)	(0.418)	(0.420)	(0.515)	(0.735)
TR		0.768	−0.279	−1.043	4.321**	1.276
		(1.448)	(1.513)	(1.520)	(1.682)	(2.223)
FA		0.125***	0.094***	0.086***	0.116***	0.199***
		(0.026)	(0.026)	(0.026)	(0.029)	(0.049)
Ul		−0.007***	−0.007***	−0.008***	−0.002***	−0.007***
		(0.000)	(0.001)	(0.001)	(0.001)	(0.001)
ER		0.013***	0.011***	0.012***	0.017***	0.008**
		(0.003)	(0.003)	(0.003)	(0.003)	(0.004)
Year FE	Yes	Yes	Yes	Yes	Yes	Yes
City FE	Yes	Yes	Yes	Yes	Yes	Yes
Constant	1.416***	3.907***	2.405***	2.082**	5.070***	4.612***
	(0.026)	(0.848)	(0.830)	(0.835)	(0.991)	(1.445)
*N*	3,488	3,488	2,896	2,896	2,224	1,856
*R* ^2^	0.002	0.752	0.764	0.761	0.715	0.773

Standard errors in parentheses; ***p < 0.01, **p < 0.05, *p < 0.1.

To further investigate the synergistic effects of the dual-pilot policy, this study conducts a comparative analysis. When assessing the impact of single-pilot policies, the experimental group consists of cities that have implemented either the Innovative City or Smart City pilot program, with non-pilot cities serving as the control group. For the dual-pilot policy, the experimental group includes cities that have implemented both pilots simultaneously, while non-pilot and single-pilot cities serve as control groups. The results in columns (3) through (6) of Table 2 show that both single-pilot and dual-pilot policies significantly improve work-safety governance outcomes. Specifically, compared to non-pilot cities, the number of work-safety accident fatalities decreases by an average of 57.6 in Innovative City single-pilot cities, 36.7 in Smart City single-pilot cities, and 69.8 in dual-pilot cities. Furthermore, compared to single-pilot cities, dual-pilot cities experience an average reduction of 38.8 fatalities. This comparative analysis demonstrates that dual-pilot cities achieve more significant improvements in work-safety governance than both non-pilot and single-pilot cities. This supports the conclusion that the combined policies produce a “1 + 1 > 2” synergistic effect, playing a crucial role in enhancing urban public safety.

This study also investigates the differential impact of the policy implementation sequence on work-safety governance outcomes, focusing on 37 cities that adopted the dual-pilot policy. Among these, 16 cities were first approved as Innovative City pilots, followed by Smart City pilot approval (referred to as “Innovation-First Cities”); 18 cities received Smart City pilot approval first, followed by Innovative City pilot approval (“Smart-First Cities”); and 3 cities obtained approval for both pilots simultaneously. Excluding the latter group, we examined the sequence effect through two regression analyses. First, using non-pilot cities as the control group, we compared the effects of “Innovation-First” and “Smart-First” cities as experimental groups, with results presented in Panel A of Table 3. Second, using single-pilot cities as the control group, we performed a similar analysis, shown in Panel B of Table 3. The findings reveal that the synergistic effect of the “Innovation-First” sequence is significantly stronger than that of the “Smart-First” sequence. A possible explanation is that Innovation-First Cities, by establishing systematic institutional innovations, optimizing industrial structures, fostering technological R&D, accumulating talent, and cultivating an innovation culture, have developed a more comprehensive innovation ecosystem. This ecosystem provides a robust foundation, both in “soft environments” and “hard capabilities,” for subsequent Smart City construction. In contrast, Smart-First Cities, despite prioritizing information infrastructure deployment, often lack complementary institutional frameworks, industrial ecosystems, talent pools, and governance cultures. As a result, their technological applications tend to remain at a basic tool level, limiting deep integration with urban governance systems and, ultimately, constraining the synergy effect on work-safety governance. Therefore, the policy implementation sequence matters: prioritizing the development of a mature innovation production system creates a stronger institutional and application environment, enabling subsequent technology empowerment to unleash greater policy effectiveness.

**Table 3:** Comparison by implementation sequence.

Variables	Panel A		Panel B	
	Innovation-First	Smart-First	Innovation-First	Smart-First
Dual_policy	−0.937***	−0.657***	−0.572***	−0.320**
	(0.100)	(0.096)	(0.129)	(0.127)
Control variables	Yes	Yes	Yes	Yes
Year FE	Yes	Yes	Yes	Yes
City FE	Yes	Yes	Yes	Yes
Constant	4.211***	4.197***	1.311	3.425**
	(1.026)	(0.912)	(1.622)	(1.446)
Observations	1,888	1,920	1,520	1,552
*R* ^2^	0.707	0.703	0.783	0.785

Standard errors in parentheses; ***p < 0.01, **p < 0.05, *p < 0.1.

### Parallel trend test

**Figure 2:**
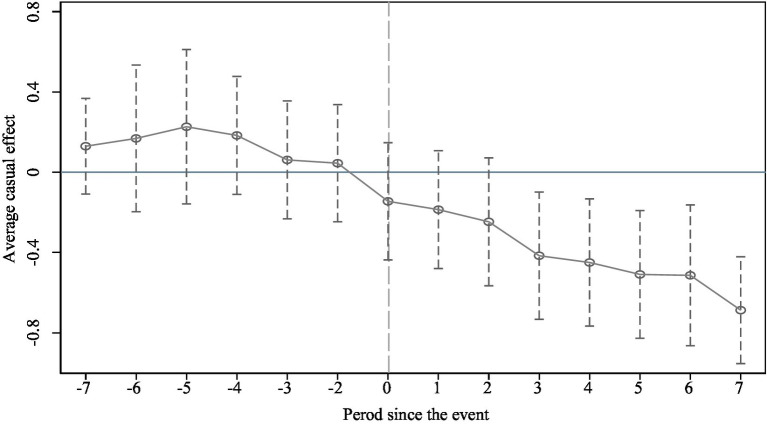
Parallel-trends test results.

The validity of the DID model hinges on the parallel trends assumption, which states that, absent the policy intervention, work-safety governance outcomes in pilot and non-pilot cities should follow similar trajectories over time. Any significant divergence in outcomes after the policy implementation can thus be attributed to the policy’s effect. To test this, the study employs an event study approach, using the year of dual-pilot policy implementation as the baseline and excluding pre-implementation periods to avoid multicollinearity. This method assesses the dynamic impact of the policy over an eight-year window before and after implementation. As shown in Figure 2, the estimated coefficients for periods prior to policy implementation are positive but statistically insignificant, indicating no significant pre-policy differences between pilot and non-pilot cities, thereby satisfying the parallel trends assumption. However, beginning 2 years after implementation, the coefficient for the dual-pilot policy becomes significantly negative and continues to decrease, signaling a substantial and sustained improvement in work-safety governance attributable to the policy. Notably, while the coefficient trend spans 14 years, statistically significant effects only emerge after a two-year lag. This delay aligns with the nature of China’s pilot policy mechanisms. As progressive reform tools, pilot programs impose strong incentives and prerequisites on selected cities. In anticipation of qualification, local governments often undertake preparatory actions aligned with selection criteria, causing a pre-implementation dip in coefficients. Yet, constrained by limited resources before formal support, initial investments in innovation and smart city development tend to be modest, delaying measurable outcomes. It is only after receiving substantial policy backing and funding from the central government, coupled with approximately 2 years of systematic construction and implementation, that pilot cities begin to exhibit significant advancements in work-safety governance.

### Robustness checks

#### Placebo test

**Figure 3:**
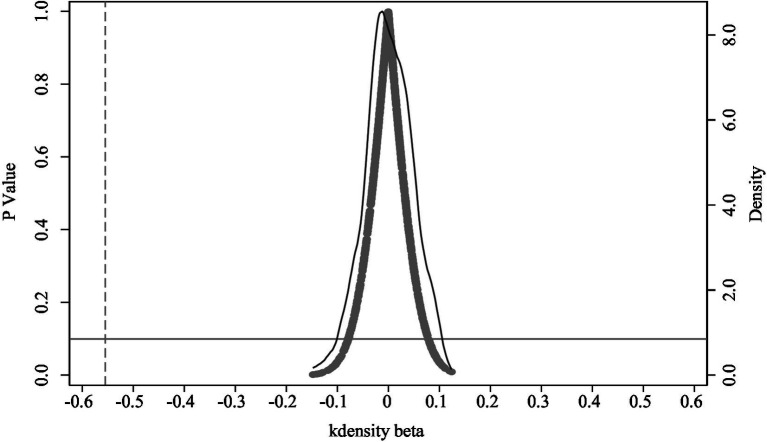
Placebo-test results.

To ensure robustness and address potential omitted-variable bias, this study conducts a placebo test. Specifically, a random subset of the 218 cities is repeatedly (1,000 times) assigned as the “treatment” group, with the remainder serving as the control group. This randomization tests whether the observed governance effect attributed to the dual-pilot policy persists under arbitrary groupings. If the dual-pilot coefficient remained significant under random assignments, it would suggest that unobserved factors, not the policy, drive the observed effect. Conversely, if coefficients from these placebo tests are mostly insignificant, it supports the conclusion that the policy effect is genuine. The simulation results show that coefficients from random assignments cluster near zero, with the majority of *p*-values exceeding 0.1. In contrast, the baseline regression yields a dual-pilot coefficient of −0.554 (represented by the dashed line in Figure 3), which clearly stands apart from the placebo distribution as an outlier. This provides strong evidence that the observed positive impact of the dual-pilot policy on work-safety governance is not confounded by omitted-variable bias.

#### Instrumental-variables approach

Against the backdrop of a national governance paradigm that seeks to “secure high-level safety through high-quality development and promote high-quality development through high-level safety,” Chinese cities are accelerating a shift toward technology enablement, intelligence-driven operations, and refined governance to effectively prevent and reduce work safety accidents, casualties, and economic losses. This transformation may be endogenously related to work-safety governance outcomes, suggesting potential bidirectional causality. To address endogeneity concerns, this study employs the number of regular higher education institutions at the city level as an instrumental variable (IV) and estimates the model using a two-stage least squares (2SLS) framework.

The rationale is twofold: first, the number of higher education institutions strongly correlates with the stock of high-level human capital, which underpins TI and the shift toward intelligent, digital transformation, thereby increasing a city’s likelihood of being selected for the Innovative City and Smart City dual pilot programs. Second, the number of such institutions is largely determined by long-term factors, such as geographic location, natural endowments, political status, and economic development level, and is plausibly unrelated to the city’s work safety accident incidence, thus satisfying the exclusion restriction. As shown in Table 4, column (1), the coefficient on the IV is positive and highly significant at the 1% level, confirming its strong relevance to the dual-pilot policy. The first-stage F-statistic is well above the conventional threshold of 10, and the Kleibergen-Paap rk LM test rejects under-identification at the 5% level, alleviating concerns of weak instruments. In the second stage [Table 4, column (2)], the 2SLS estimates reveal that the dual-pilot policy coefficient remains negative and statistically significant at the 1% level. These results confirm that the core findings of the study hold even after accounting for potential endogeneity and bidirectional causality.

**Table 4:** Robustness check results A.

Variables	2SLS		PSM—DID	Replace the explained variable
	First stage	Second stage		
	(1)	(2)	(3)	(4)
Dual_policy		−5.1435***	−0.679*	−0.054***
		(0.925)	0.401	(0.009)
IV	0.012***			
	(0.002)			
Control variables	Yes	Yes	Yes	Yes
Year FE	Yes	Yes	Yes	Yes
City FE	Yes	Yes	Yes	Yes
Constant			10.111*	1.683***
			(6.530)	(0.097)
*N*	3,488	3,488	956	3,488
*R* ^2^			0.810	0.718
The F statistic of the first stage	47.07			
Kleibergen-Paap rk LM statistic		104.920**		
Kleibergen-Paap Wald rk F statistic		50.230 [16.380]		

Standard errors in parentheses; ***p < 0.01, **p < 0.05, *p < 0.1.

#### Propensity score matching combined with DID test

To mitigate endogeneity arising from sample self-selection, this study applies Propensity Score Matching combined with DID (PSM–DID) as a robustness check on the baseline estimates. Specifically, control variables serve as matching covariates using caliper nearest-neighbor matching, and the resulting matched sample is then analyzed through DID estimation. As shown in Table 4, column (3), the coefficient for the dual-pilot policy remains negative and statistically significant at the 5% level, thereby confirming the robustness of the baseline results.

#### Alternative dependent variable

We replace the dependent variable with the death rate from work safety accidents per 100 million yuan of GDP and re-estimate the effect of the dual-pilot policy on work safety governance. The results, presented in Table 4, column (4), show that the coefficient for the dual-pilot policy remains negative and statistically significant, thereby confirming the robustness of the baseline findings.

#### Eliminate the interference of other policies during the same period

For a rigorous identification of the net effect of the dual-pilot policy on work safety governance, it is crucial to control for confounding influences from other policies implemented during the study period. A review identified several such policies potentially affecting work safety governance, including Made in China 2025, Broadband China, and Supervised Listing (guapai duban). These policies were incorporated as control variables to isolate the specific impact of the dual-pilot policy. The results shown in Table 5, columns (1)–(4), indicate that while the coefficients for Made in China 2025, Broadband China, and Supervised Listing are each significantly negative, reflecting reductions in fatalities, the coefficient for the dual-pilot policy remains negative and statistically significant at the 1% level even after controlling for these policies. This provides strong evidence that the observed improvements in work safety governance can be robustly attributed to the Innovative City + Smart City dual-pilot policy, independent of other concurrent policy effects.

**Table 5:** Robustness check results B.

Variables	Eliminate other policy interferences				The explained variable lags behind by two phases
	(1)	(2)	(3)	(4)	(5)
Dual_policy	−0.507***	−0.524***	−0.538***	−0.477***	−0.542***
	(0.074)	(0.076)	(0.076)	(0.075)	(0.083)
Made in China 2025	−0.889***			−0.872***	
	(0.082)			(0.083)	
Broadband China		−0.126**		−0.067	
		(0.053)		(0.052)	
Place it under supervision			−0.133***	−0.122**	
			(0.050)	(0.049)	
Control variables	Yes	Yes	Yes	Yes	Yes
Year FE	Yes	Yes	Yes	Yes	Yes
City FE	Yes	Yes	Yes	Yes	Yes
Constant	3.934***	3.922***	3.864***	3.901***	5.828***
	(0.833)	(0.847)	(0.847)	(0.833)	(1.001)
*N*	3,488	3,488	3,488	3,488	3,052
*R* ^2^	0.760	0.752	0.752	0.761	0.784

Standard errors in parentheses;***p < 0.01, **p < 0.05, *p < 0.1.

#### The explained variable lags behind by two phases

Considering the observed two-year lag in the policy effect, the model was re-estimated using a dependent variable lagged by two periods. The results, reported in Table 5, column (5), show that the coefficient for the dual-pilot policy remains negative and statistically significant at the 1% level. This finding further confirms that the combined implementation of the Innovative City and Smart City policies significantly enhances work safety governance, reinforcing the earlier conclusions.

### Mechanism analysis

#### Technological-progress effect

The dual-pilot policy significantly enhances work safety governance through technological progress. As shown in Table 6, column (1), the policy exerts a positive and statistically significant impact on technological advancement at the 1% level, indicating its role as a crucial driver of innovation. The continuous development of emerging technologies, such as emergency-response equipment, industrial robots, automated production, intelligent monitoring and early-warning models based on big data, and IoT-enabled equipment monitoring, has increasingly enabled the substitution of human labor in high-risk environments. This reduces manual intervention, strengthens risk prevention and control, optimizes emergency response, and substantially improves intrinsic safety, thereby effectively curbing the occurrence of work safety accidents. Thus, Hypothesis H2 is supported.

**Table 6:** Mechanism results.

Variables	TI	Safety	IU	Safety	Safety	Safety
	(1)	(2)	(3)	(4)	(5)	(6)
Dual_policy	0.248***	−0.593***	−0.008**	−0.567***	−0.554***	−0.541***
	(0.042)	(0.076)	(0.004)	(0.075)	(0.075)	(0.076)
TI		−0.153***				
		(0.031)				
IU				1.575***		
				(0.375)		
SR					−17.675*	−17.628*
					(9.477)	(9.470)
Dual_policy × Safety Regulation						−80.622**
						(32.715)
Control variables	Yes	Yes	Yes	Yes	Yes	Yes
Year FE	Yes	Yes	Yes	Yes	Yes	Yes
City FE	Yes	Yes	Yes	Yes	Yes	Yes
Constant	9.505***	2.449***	−0.771***	2.693***	4.010***	4.001***
	(0.472)	(0.896)	(0.040)	(0.894)	(0.850)	(0.849)
*N*	3,488	3,488	3,488	3,488	3,488	3,488
*R* ^2^	0.934	0.754	0.906	0.753	0.752	0.752

Standard errors in parentheses; ***p < 0.01, **p < 0.05, *p < 0.1.

To fully realize the governance benefits of technological progress, local governments should provide comprehensive policy support across the three stages of “R&D – Application – Integration.” During the R&D stage, special funds should be established to support key safety technologies such as industrial robots, intelligent sensors, and emergency warning models. In the application stage, governments should encourage enterprises, particularly SMEs and high-risk firms, to adopt safety emergency equipment through incentives like tax reductions and subsidies, while promoting the mechanization, automation, and intelligent transformation of hazardous production activities. In the integration stage, collaborative innovation platforms that connect government, industry, academia, research, and application (“Gov-Industry-Academia-Research-Application”) should be fostered. Additionally, unified data interface and system integration standards should be set to promote interoperability among intelligent monitoring systems and to build an intelligent safety production sensing network spanning the entire industrial chain. This holistic approach will elevate technological empowerment from isolated breakthroughs to systemic enhancement.

#### Industrial-upgrading effect

The dual-pilot policy promotes work safety governance through industrial upgrading. As shown in Table 6, column (3), the policy has a significant negative effect on industrial upgrading at the 1% significance level, demonstrating that policy-driven industrial transformation helps reduce the likelihood of accidents. By advancing smart manufacturing and automation, optimizing production processes and organizational structures, enhancing production efficiency, and minimizing manual operations, the policy provides systemic support across multiple dimensions, including safety concepts, institutional norms, and technical equipment, jointly driving improvements in work safety governance. Thus, Hypothesis H3 is supported.

To steer industrial upgrading toward intrinsic safety, the government should prioritize strategic coordination between industrial and safety policies. First, differentiated industrial guidance strategies should be implemented by raising standards for safety, environmental protection, and energy consumption, while strictly controlling or gradually phasing out high-risk industries. Concurrently, subsidies should support traditional enterprises undertaking safety, intelligent, and automation transformations. Second, the development of a safety industry ecosystem should be emphasized. When planning emerging industrial clusters and parks, shared safety infrastructure, such as regional risk monitoring centers, specialized emergency rescue stations, and common technology service platforms, should be mandated, facilitating centralized investment in safety capabilities and fostering co-building and sharing concepts. Third, the government should promote an integrated “industry–talent–safety” policy package by including safety management professionals, intelligent operation and maintenance engineers, and other critical talent in shortage talent directories, supported by policies for talent recruitment and training. This will build a strong safety-oriented human capital foundation to underpin industrial upgrading.

#### Safety-regulation effect

SR plays a significant moderating role in enhancing the effects of work-safety governance under the dual-pilot policy. As shown in Table 6, column (5), SR has a significant negative effect on work-safety accident outcomes, indicating its effectiveness in preventing and curbing safety incidents. Column (6) further reveals that the interaction term between the dual-pilot policy and SR (Dual_policy × SR) has a coefficient of −80.622, significant at the 5% level, suggesting that SR amplifies the positive impact of the dual-pilot policy on governance outcomes. This synergy likely arises because traditional SR, which primarily focuses on “human defense,” has been organically integrated with the “technology defense + joint defense” model promoted by the dual-pilot policy. Together, they form a collaborative governance framework combining “human defense + technology defense + joint defense,” enabling timely identification and elimination of safety hazards. Specifically, SR raises standards, strengthens enforcement, and increases penalties for violations, compelling enterprises to strictly comply with safety protocols, increase safety investments, and adopt advanced production technologies, thus establishing the hardware and institutional foundation for the “technology defense + joint defense” model. Meanwhile, intelligent platforms and cross-departmental cooperation networks established by the dual-pilot policy enhance safety supervision’s precision and efficiency through information sharing and collaborative governance mechanisms. Based on these findings, hypotheses H4a and H4b are supported.

To further strengthen government regulation, it is essential to accelerate the construction of a modern SR system. First, local governments should deepen the digital transformation of work-SR by actively adopting digital regulatory tools such as IoT inspections and big data analytics. These technologies can address limitations of traditional “human defense” approaches by enhancing regulatory coverage and enabling real-time dynamic monitoring, thereby fostering a modern regulatory framework that integrates online monitoring and early warning with offline precise governance through coordinated actions. Second, an incentive-compatible regulatory mechanism should be established. While maintaining stringent enforcement, governments should link enterprises’ work-safety credit status to enforcement inspection frequency, workers’ compensation insurance rates, tax incentives, and credit support, creating a market-driven incentive structure of “compliance rewards and violation penalties.” This encourages enterprises to move from passive compliance toward proactive investment in safety. Third, regulatory legislation should accelerate the adoption of technology-enabled tools. Institutional frameworks should convert proven risk-warning models, intelligent diagnostic algorithms, and other TIs into standardized, scalable regulatory instruments to ensure hazard identification, law enforcement supervision, and emergency decision-making rely on real-time, comprehensive, and accurate data, thereby systematically enhancing the deterrence and credibility of SR.

### Spatial spillover effects

The coordinated implementation of the dual pilot policy not only facilitates the optimization of production processes by local enterprises but also acts as a catalyst for enhancing operational efficiency, enabling automated production, and fostering intelligent risk prevention and control, thereby improving intrinsic safety levels. Additionally, by leveraging increasingly advanced transportation and information networks, the policy accelerates the inter-city flow of knowledge and technology, generating spatial spillover effects on work safety governance in neighboring cities. Drawing on Liu et al., this study employs a spatial durbin model (SDM) to examine whether the dual pilot policy produces significant spatial spillover effects.


$$\begin{array}{l} \text{Safet}y_{\mathrm{it}}=\alpha_{0}+\rho \sum_{1}^{n}w_{\mathrm{ij}}\text{Safet}y_{\mathrm{it}}+\alpha_{1}\text{Dual}\_\text{polic}y_{\mathrm{it}}\\ +\alpha_{2}\sum_{1}^{n}w_{\mathrm{ij}}\text{Dual}\_\text{polic}y_{\mathrm{it}}+\alpha_{3}\sum_{1}^{n}X_{\mathrm{ij}}\\ +\alpha_{3}\sum_{1}^{n}w_{\mathrm{ij}}X_{\mathrm{ij}}+\mu_{i}+t_{i}+\varepsilon_{\mathrm{it}} \end{array}$$ (5)

In Equation 5, *ρ* represents the spatial autoregressive coefficient, and *w_ij_* denotes the spatial weight matrix. Initially, this study employs an economic-geographic distance spatial weight matrix for preliminary regression analysis, followed by the use of an inverse-distance-squared spatial weight matrix to test the robustness of the estimates. Given that spatial dependence typically diminishes with increasing distance, spatial spillover effects often exhibit a distance boundary. To examine the effective range of these spillovers, we further construct an inverse-distance spatial weight matrix and perform segmented regressions with 100-kilometer intervals.

The empirical analysis is conducted using a two-way fixed-effects SDM. Table 7 reports the spatial spillover effects of the dual pilot policy along with their decomposition. The results show a significantly negative direct effect, consistent with baseline regressions that demonstrate improvements in local work safety governance. The indirect effect is also significantly negative, indicating beneficial spillover effects that reduce accident fatalities in neighboring cities. Consequently, the total effect is significantly negative, confirming that the dual pilot policy substantially lowers the incidence of work safety accidents and casualties overall. These findings highlight that the modern urban construction paradigm of “innovation-driven + digital enablement” effectively enhances urban safety resilience.

**Table 7:** Spatial-spillover regression results.

Variables	(1)	(2)
Dual_policy	−0.556***	−0.511***
	(0.071)	(0.068)
W × Dual_policy	−3.866***	−1.441***
	(0.726)	(0.279)
Direct effect	−0.595***	−0.558***
	(0.073)	(0.072)
Indirect effect	−9.742***	−2.332***
	(2.476)	(0.768)
Total effect	−10.337***	−2.890***
	(2.491)	(0.794)
Control variables	Yes	Yes
Year FE	Yes	Yes
City FE	Yes	Yes
Rho	0.551***	0.654***
	(0.077)	(0.033)
Sigma2_e	0.500***	0.456***
	(0.012)	(0.011)
*N*	3,488	3,488
*R* ^2^	0.028	0.005

Standard errors in parentheses; ***p < 0.01, **p < 0.05, *p < 0.1.

**Figure 4:**
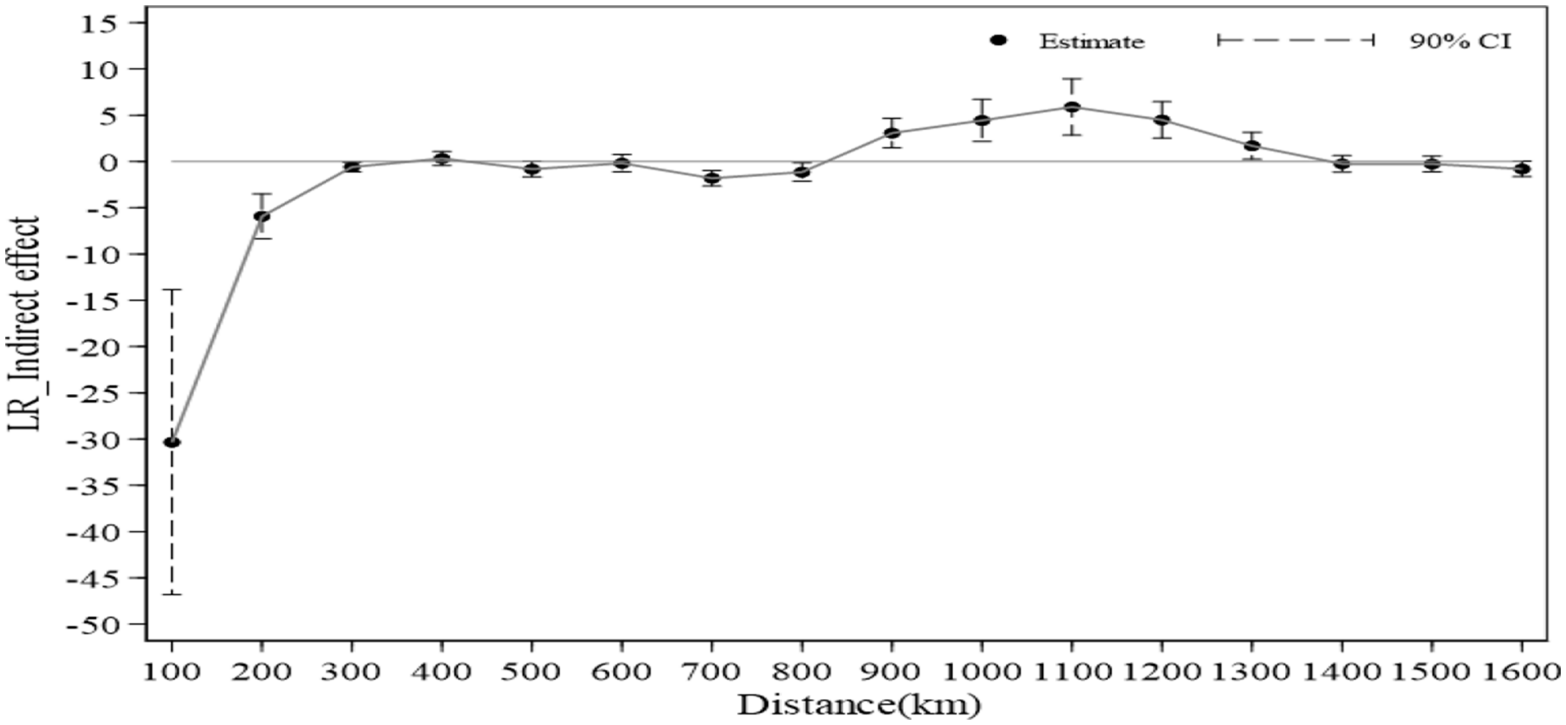
The spatial distance spillover effect of the dual pilot policy.

This study conducts multi-interval regressions at 100 km increments, estimating the policy effects and their 90% confidence intervals at each distance threshold. Based on these results, a spatial spillover effect decay boundary is plotted in Figure 4, revealing a distinct three-stage pattern in how the dual-pilot policy effects vary with geographic distance.

Within 800 km: The establishment of dual-pilot cities generates a significant positive spillover effect on work-safety governance in neighboring cities. However, the intensity of this effect weakens as the distance increases. This is primarily because the innovative models and governance experiences cultivated in dual-pilot cities are shared and emulated by surrounding cities via trade interactions and interregional cooperation among officials. Mechanisms such as knowledge exchange, resource matching, and experience learning facilitate this effective diffusion. Additionally, non-pilot cities nearby tend to adopt successful pilot city practices to avoid central government accountability for safety incidents, resulting in a “Golden Forest Learning Effect,” whereby they obtain feasible governance solutions at lower costs.

Between 800 km and 1,400 km: In this intermediate range, the dual-pilot policy exhibits a significant negative spatial spillover effect on surrounding cities’ work-safety governance. Here, competitive effects outweigh positive knowledge spillovers. Pilot cities leverage their institutional advantages to create a “siphoning effect,” attracting vital resources such as specialized investments, technical talent, and high-quality safety enterprises away from neighboring regions, thereby depleting their capacity to improve safety governance. Concurrently, due to political promotion pressures and the “one-vote veto” mechanism on safety assessments, non-pilot city governments in this zone engage in “benchmark competition.” Their imitation efforts often prioritize visible safety equipment installation over systemic governance reforms, with some diverting routine safety supervision resources to highlight projects. This leads to structural suppression of their overall safety governance effectiveness.

Beyond 1,400 km: When distances exceed 1,400 km, the number of spatially connected cities sharply decreases, causing the spatial spillover estimates for the dual-pilot policy to fluctuate randomly and lose statistical significance. This suggests that at such distances, inter-city dependencies are too weak to be captured effectively by the model.

In summary, the spatial impact of dual-pilot city construction on work-safety governance follows a clear distance decay pattern of “near-field promotion, mid-to-far-field suppression, far-field disappearance,” consistent with the First Law of Geography’s principle that spatial correlation diminishes with distance.

### Heterogeneity analysis

#### City type

Drawing on the classification criteria in the National Plan for the Sustainable Development of Resource-Based Cities (2013–2020), the empirical analysis divides the sample into resource-based and non-resource-based cities. The regression results in Table 8, columns (1)–(2), show that the dual pilot policy significantly enhances work safety governance in both city types, with notably stronger effects observed in non-resource-based cities. This heterogeneity likely arises from the structural rigidity inherent in resource-based economies, which remain heavily dependent on natural resources and are dominated by high-risk, resource-intensive industries such as mining, metallurgy, and chemical production. The associated safety risks tend to be highly physical, complex, and hazardous, for example, mine collapses and underground gas explosions, requiring governance approaches reliant mainly on engineering protections and manual expertise. Additionally, the unique production environments in these sectors, such as underground mining and high-temperature or high-pressure workshops, limit the adaptability of smart technologies like the IoT and real-time monitoring. Challenges including signal interference, equipment corrosion, and high installation costs substantially raise the barriers for technology deployment. These technical constraints, combined with path dependence on resource extraction that dampens local innovation incentives, limited safety investments, weak law enforcement, and relatively low worker skills and safety awareness, severely hinder the transformation of the dual pilot policy’s synergistic potential into concrete improvements in work safety governance.

**Table 8:** Results of heterogeneity test.

Variables	City type		City size		City region		
	Resources-based	Non-resources-base	Small/medium-sized	Large size	Eastern	Central	Western
	(1)	(2)	(3)	(4)	(5)	(6)	(7)
Dual_policy	−0.296***	−0.587***	−0.530***	−0.412***	−0.572***	−0.912***	−0.107
	(0.103)	(0.103)	(0.139)	(0.082)	(0.133)	(0.138)	(0.098)
Control variables	Yes	Yes	Yes	Yes	Yes	Yes	Yes
Year FE	Yes	Yes	Yes	Yes	Yes	Yes	Yes
City FE	Yes	Yes	Yes	Yes	Yes	Yes	Yes
Constant	4.135***	0.798	−0.516	3.156***	10.993***	−0.446	2.381**
	(0.829)	(1.453)	(2.315)	(0.782)	(1.889)	(1.294)	(1.168)
*N*	1,440	2,048	2,224	1,264	1,264	1,264	960
*R* ^2^	0.689	0.760	0.778	0.739	0.791	0.707	0.754

Standard errors in parentheses; ***p < 0.01, **p < 0.05, *p < 0.1.

In contrast, non-resource-based cities typically feature more diversified industrial structures characterized mainly by soft risks, such as equipment failures, operational errors, managerial negligence, and hazards related to public infrastructure, that are more malleable and manageable. The intelligent production and regulatory technologies promoted by the policy have broader applicability across various settings including factories, retail spaces, roads, and buildings, and generally involve lower deployment thresholds and adaptation costs. Moreover, non-resource-based cities often exhibit higher degrees of marketization and more flexible governance frameworks. Enterprises and institutions in these cities tend to demonstrate stronger responsiveness to policy incentives, greater capacity to absorb new technologies and management models, and a workforce with relatively better digital literacy. These factors collectively reduce the costs and resistance associated with management transformation and workforce reskilling. Consequently, in environments characterized by greater institutional flexibility and more adaptable risk structures, the policy’s technological dividends can be more fully realized, resulting in a more pronounced enhancement of work safety governance.

#### City size

Based on the classification system defined in the State Council’s 2014 Notice on Adjusting the Criteria for City Size Classification, the sample is divided into small/medium-sized and large cities for separate regression analyses. The results indicate that the dual pilot policy significantly improves work safety governance in both categories, with a more pronounced effect observed in small and medium-sized cities. This stronger impact may be due to the relatively lower baseline governance capacity in these cities, coupled with greater potential for progress in infrastructure development, technological adoption, and refined governance practices. The innovation resources, advanced governance concepts, and intelligent technologies introduced by the dual pilot policy can more effectively address existing gaps, leading to faster and more visible improvements in work safety governance in small and medium-sized cities.

#### City region

To explore the regional heterogeneity of the dual-pilot policy effects, this study divides China into three major regions, East, Central, and West, based on geographic, economic, political, and cultural differences. The results presented in columns (5) to (7) of Table 8 indicate that the dual-pilot policy exerts differential impacts on work-safety governance across these regions, with the strongest effect observed in the Central region, followed by the East region, and no significant effect detected in the West region. This disparity closely aligns with the varying developmental stages and infrastructure conditions of each region. The Central region, currently undergoing accelerated industrialization, benefits directly from the technological empowerment and governance transformation driven by the dual-pilot policy, which contribute to the modernization of its local work-safety governance systems and capabilities. Moreover, the Central region’s relatively well-established industrial base and sufficient innovation resources enable it to efficiently absorb and convert policy inputs from the pilot programs into substantial improvements in safety governance. In the East region, where work-safety governance systems are already relatively mature, the policy’s effects are more marginal, representing incremental improvements. However, the region’s advanced digital infrastructure and robust innovation ecosystem provide strong support for policy implementation, facilitating continuous enhancements in governance outcomes. In contrast, the West region faces significant challenges stemming from overall weaker infrastructure, particularly limited digital access, sparse coverage of smart facilities, and less developed industrial support capabilities. These constraints pose substantial barriers to the implementation of innovation policies and the adoption of new-generation information technologies. The infrastructure gap, shaped by economic development levels and regional characteristics, hinders the innovation-driven and smart-empowered mechanisms central to the dual-pilot policy, resulting in a lack of significant policy impact in the West. This finding aligns closely with existing research (52), which highlights how spatial imbalances can suppress policy implementation outcomes (Table 8).

### Research conclusions and policy recommendations

#### Research conclusions

Based on panel data from 218 prefecture-level cities in China (2008–2023) and treating the dual pilot policy as a quasi-natural experiment, this study employs a multi-period DID model to assess the policy’s impact on safety governance. The main findings are as follows: (1) The dual pilot policy significantly improves governance, with synergy effects outperforming both non-pilot and single-pilot cities. Moreover, the sequence of “innovation first, then smart” proves more effective than the reverse order. (2) Mechanism tests reveal technological progress and industrial upgrading as key channels, with SR positively moderating the policy’s effect. (3) Spatial spillover analysis shows promotion effects within 800 km, suppression between 800 and 1,400 km, and dissipation beyond 1,400 km, indicating an attenuation boundary. (4) Heterogeneity analysis finds stronger policy effects in non-resource-based cities, small and medium-sized cities, and cities in the central region.

From a public health perspective, these findings highlight the alignment between China’s work-safety governance initiatives and the United Nations SDGs, particularly SDG 3 (Good Health and Well-being) and SDG 8 (Decent Work and Economic Growth). On one hand, work-safety serves as a crucial link connecting economic development with the safety and health of the workforce. The dual-pilot policies validated by this study reduce the incidence of work-safety accidents through technological advancement and industrial upgrading, thereby effectively safeguarding workers’ lives, health, and safety. Each avoided accident represents a decrease in potential casualties, contributing significantly to SDG 3’s goal of “reducing injuries and deaths.” Simultaneously, a safe work environment is fundamental to ensuring “decent work.” When workers no longer face a trade-off between their personal safety and employment, economic growth embodies inclusivity and sustainability, truly reflecting a “people-centered” approach. This constitutes a strong response to SDG 8. On the other hand, the digital transformation of SR is fostering a public governance system that promotes occupational health. The “human defense + technology defense + joint defense” governance framework, built through innovation-driven strategies, smart technologies, and traditional SR, not only helps prevent sudden safety incidents but also leverages technology to automate and enable unmanned operation of hazardous tasks. This reduces the incidence of occupational diseases caused by prolonged exposure to dust, toxic substances, and ergonomic risks. This represents a shift in work-safety governance from “end-of-pipe management,” which focuses on accident response, to “source governance” and “process governance,” which aim to proactively prevent occupational health risks. This evolution reflects the deep integration of public health principles into work-safety governance. In conclusion, China’s Innovative City and Smart City pilot programs in modern urban development extend beyond TI and economic growth. At their core, they constitute large-scale public health interventions designed to protect and promote human safety and well-being. By creating safer, smarter, and more sustainable production systems, these policies translate economic development outcomes into tangible guarantees for human life safety and physical health. They provide strong evidence and practical pathways from China’s urban governance practice for achieving SDG 3 and SDG 8.

#### Policy recommendations

First, strengthen policy coordination and optimize sequencing. Given the superior effectiveness of dual pilot programs over single policies, governments should systematically integrate resources across initiatives such as Innovative City, Smart City, and Resilient City. Establishing a dynamic synergy-optimization mechanism can help overcome policy fragmentation and achieve complementarity in intensity, scope, and depth. In practice, eligible single-pilot cities should be upgraded to dual or multi-pilot status to unleash multiplier effects. Sequencing should be planned scientifically: prioritize building innovation capacity first by establishing full-chain ecosystems, from basic research to breakthroughs, to technology transfer, particularly targeting intelligent risk identification and accident simulation bottlenecks. This will lay a solid safety foundation for Smart City development. Subsequently, empower services, management, and safety systems using AI, big data, IoT, and blockchain technologies to create a closed loop from R&D to intelligent prevention and control.

Second, build a tiered spatial co-governance system. Based on the observed attenuation patterns, establish regional co-governance within core radiation circles. Provincial governments can facilitate cross-jurisdiction work safety agreements that clarify joint prevention and control responsibilities and resource allocation authority. They can also co-develop emergency stockpiles and shared early-warning platforms to enhance the spatial transmission of policy benefits. Innovate benefit coordination by earmarking a portion of core cities’ safety-related tax revenues into special compensation funds, which can be used to purchase risk-assessment and training services from surrounding cities, thereby offsetting externalities from knowledge spillovers. Support leading cities in setting up collaborative innovation centers for work safety and hosting regular exchange and capacity-building events. For peripheral zones where effects attenuate, adopt cloud-based technical coordination and remote expert systems with shared supervision platforms to efficiently transfer management experience and technical solutions, addressing resource constraints. This approach will foster a tiered, coordinated regional safety development pattern.

Third, improve the SR system and strengthen enforcement. Develop a comprehensive three-in-one mechanism encompassing standards setting, law enforcement supervision, and capacity building. Accelerate the formulation of standards for AI and big data applications in high-risk scenarios, such as intelligent monitoring of hazardous operations and IoT device operation and maintenance. Promote data sharing, intelligent supervision, and mandatory open safety data interfaces to break information silos and enable dynamic risk identification and precision enforcement. Enhance frontline regulatory teams through professional training, equipment upgrades, and policy incentives to comprehensively improve enforcement effectiveness.

Fourth, adopt differentiated development strategies. Non-resource-based cities should leverage their diversified industrial bases and strong innovation capacities to build safety-technology incubators, reinforced through R&D tax incentives, accelerating the diffusion of emerging technologies into safety production. This will facilitate the co-evolution of low-risk industries alongside advanced safety-control systems. Resource-based cities must overcome path dependence by establishing special funds for digital and intelligent retrofits in high-risk sectors such as mining and chemicals to mitigate systemic risks. Regarding city size, fiscally constrained small and medium-sized cities can promote lightweight smart safety solutions, such as low-cost sensors combined with cloud platforms, to reduce costs and improve efficiency, while large cities should develop system-level risk prevention frameworks and cross-department platforms for major hazard supervision. Regionally, the eastern region should focus on key technology breakthroughs and serve as a hub for standards development and diffusion; the central region should amplify policy synergies; and the western region should receive targeted infrastructure investments and precision policy support, including east–west partnerships for technology transfer and capacity building to foster endogenous governance capabilities.

#### Limitations and future studies

The limitations of this study are primarily reflected in three key aspects. First, the limitation of the dependent variable. This study uses the number of fatalities from accidents as a proxy for work-safety governance effectiveness. While this indicator is direct and objective, it does not fully capture the multifaceted nature of governance effectiveness. For example, it fails to account for the frequency or severity of accidents and does not reflect the efforts and achievements of enterprises in areas such as risk prevention, hazard identification, safety training, and related activities. To address this limitation, future research could incorporate input factors like risk prevention, hazard identification, safety training, and safety investment, alongside economic output as the desired output, and accident frequency, severity, and fatalities as undesired outputs. Employing non-radial efficiency evaluation methods, such as the super-efficiency SBM model, may offer a more scientific and comprehensive measure of work-safety governance effectiveness. Second, the potential for omitted variable bias. Due to data availability constraints, this study did not control for micro-level variables such as job quality, workers’ safety awareness, and enterprise safety management conditions. The absence of these factors may partially compromise the accuracy of policy effect estimation. Future research that obtains more granular data on employment and labor environments would help enhance the robustness of the findings. Third, the strong reliance on administrative data. The data used in this study are derived from government public statistics and official reports, which may have systemic limitations related to measurement accuracy, statistical coverage, and timeliness. For example, some regions might experience underreporting, non-reporting, or inconsistent statistical standards, potentially introducing bias into the measurement of core variables and regression outcomes. Future studies could attempt to cross-validate findings with enterprise survey data, workers’ compensation records, and other information sources. Additionally, incorporating real-time monitoring data and online public opinion as supplementary sources may improve the reliability and validity of key variable measurements.

## Data Availability

The data that support the findings of this study are available upon request from the corresponding author. Requests to access these datasets should be directed to Peisong Han, peisonghan@163.com.
