# Correction: “Does one plus one exceed two?” The synergistic effect of Innovative City and Smart City pilots on work safety governance—evidence from a quasi-natural experiment

**DOI:** 10.3389/fpubh.2026.1775723

**Published:** 2026-01-28

**Authors:** Liqing Li, Peisong Han, Mengjie Xu

**Affiliations:** 1 College of Public Administration and Law, Hunan Agricultural University, Changsha, Hunan, China; 2 College of Sociology, Guizhou Minzu University, Guiyang, Guizhou, China

**Keywords:** Innovative City, Smart City, work safety governance, dual pilot policy, synergistic effect

There was a mistake in Table 6 as published. The estimated coefficient for the column (3), “Dual_policy”, should be negative, while the estimated coefficient for the column (4), “IU”, should have a positive sign. The corrected Table 6 appears below.

**Table 6:** Mechanism results.

**Variables**	**TI**	**Safety**	**IU**	**Safety**	**Safety**	**Safety**
	**(1)**	**(2)**	**(3)**	**(4)**	**(5)**	**(6)**
Dual_policy	0.248^***^	−0.593^***^	−0.008^**^	−0.567^***^	−0.554^***^	−0.541^***^
	(0.042)	(0.076)	(0.004)	(0.075)	(0.075)	(0.076)
TI		−0.153^***^				
		(0.031)				
IU				1.575^***^		
				(0.375)		
SR					−17.675^*^	−17.628^*^
					(9.477)	(9.470)
Dual_policy × safety regulation						−80.622^**^
						(32.715)
Control variables	Yes	Yes	Yes	Yes	Yes	Yes
Year FE	Yes	Yes	Yes	Yes	Yes	Yes
City FE	Yes	Yes	Yes	Yes	Yes	Yes
Constant	9.505^***^	2.449^***^	−0.771^***^	2.693^***^	4.010^***^	4.001^***^
	(0.472)	(0.896)	(0.040)	(0.894)	(0.850)	(0.849)
*N*	3,488	3,488	3,488	3,488	3,488	3,488
*R* ^2^	0.934	0.754	0.906	0.753	0.752	0.752

Standard errors in parentheses; ^***^p < 0.01, ^**^p < 0.05, ^*^p < 0.1.

There was a mistake in Table 8 as published. Regarding the sample data for the central and western regions in Table 8, due to an oversight in the preliminary verification process, the information previously provided contained a misstatement. We hereby issue a correction: the actual sample size for the central region is 1264, and for the western region it is 960. The corrected Table 8 appears below.

**Table 8:** Results of heterogeneity test.

**Variables**	**City type**		**City size**		**City region**		
	**Resources-based**	**Non-resources-base**	**Small/medium-sized**	**Large size**	**Eastern**	**Central**	**Western**
	**(1)**	**(2)**	**(3)**	**(4)**	**(5)**	**(6)**	**(7)**
Dual_policy	−0.296^***^	−0.587^***^	−0.530^***^	−0.412^***^	−0.572^***^	−0.912^***^	−0.107
	(0.103)	(0.103)	(0.139)	(0.082)	(0.133)	(0.138)	(0.098)
Control variables	Yes	Yes	Yes	Yes	Yes	Yes	Yes
Year FE	Yes	Yes	Yes	Yes	Yes	Yes	Yes
City FE	Yes	Yes	Yes	Yes	Yes	Yes	Yes
Constant	4.135^***^	0.798	−0.516	3.156^***^	10.993^***^	−0.446	2.381^**^
	(0.829)	(1.453)	(2.315)	(0.782)	(1.889)	(1.294)	(1.168)
*N*	1,440	2,048	2,236	1,249	1,264	1,264	960
*R* ^2^	0.689	0.760	0.778	0.739	0.791	0.707	0.754

Standard errors in parentheses; ^***^p < 0.01, ^**^p < 0.05, ^*^p < 0.1.

Table 7 and Table 8 were cited incorrectly in the article. In section **Heterogeneity analysis**, subsection *City region*, paragraph one, Table 7 has been corrected to Table 8.

Corrections have been made to the section **Policy context**, paragraphs one and two.

In paragraph one the statistical data in the sentence “By 2025, a total of 102 cities, approximately 30.63% of all cities nationwide” is incorrect. The corrected paragraph appears below:

“The Innovative City pilot policy is a cornerstone of China's innovation-driven development strategy and a key driver in transforming economic growth models. Since its inception in Shenzhen in 2008, the policy has been expanded through seven rounds of pilot projects. By 2025, a total of 97 prefecture level cities, approximately 29.13% of all cities nationwide, have been included in the pilot program. A review of policy documents reveals three defining characteristics of the Innovative City pilot policy. First, it fosters collaboration between government and market forces. Local governments coordinate the overall advancement of the initiative, while the market plays a decisive role in allocating innovation resources, creating a dynamic synergy between proactive governance and effective market mechanisms. Second, the policy aims to establish an efficient innovation production system by attracting innovative talent, increasing financial investment, strengthening protection of intellectual property, and providing comprehensive innovation platforms. These efforts continually optimize the urban innovation environment, promote the clustering of high-end industries, and comprehensively enhance cities' innovation capacity and overall competitiveness. Third, there is a strong emphasis on transforming innovative achievements into high-quality development. The policy seeks to lead in key technologies to accelerate the formation of globally competitive modern industrial systems and to promote industrial structure optimization and upgrading. Simultaneously, “safety and environmental protection” are fundamental principles, with technological progress driving the economy and society toward safe, green, low-carbon, and sustainable development.”

In paragraph two the statistical data in “encompassing 107 cities, or roughly 32.13% of the country's total cities.” is incorrect. The corrected paragraph appears below:

“The Smart City pilot policy integrates new-generation information technologies, including the IoT, cloud computing, big data, and AI, into the full spectrum of urban planning, construction, management, and services. This drives the transformation of urban governance toward greater intelligence, refinement, and modernization. The policy was officially launched in 2012 when China's Ministry of Housing and Urban-Rural Development issued the Notice on Carrying Out National Smart City Pilot Work and announced the first batch of pilot cities. Since then, China has implemented three rounds of Smart City pilots, encompassing 104 prefecture level cities, or roughly 31.23% of the country's total cities. In August 2025, the Central Committee of the Communist Party of China and the State Council issued the Opinions on Promoting High-Quality Urban Development, further underscoring Smart City construction as a core strategy to enhance urban governance effectiveness and overall capacity, with the ultimate goal of improving urban residents' sense of gain, happiness, and security.”

A correction has been made to the section **Research design**, *subsection Model construction*, paragraph one.

The statistical data in “Additionally, three batches of Smart City pilots were introduced in January 2013, August 2013, and April 2015, encompassing 163 cities.” is incorrect. The corrected paragraph appears below:

“By 2023, China had launched seven batches of Innovative City pilots (in 2008, 2010, 2011, 2012, 2013, 2018, and 2022), covering 97 prefecturelevel cities. Additionally, three batches of Smart City pilots were introduced in January 2013, August 2013, and April 2015, encompassing 104 prefecture level cities. Due to the differences in city lists and implementation timings, this study employs a staggered DID approach to estimate the impact of the dual-pilot policy on work-safety governance.”

A correction has been made to the section **Empirical results**, *subsection Industrial-upgrading effect*, paragraph one.

In this paper, we use the proportion of secondary industry output value as a proxy variable for industrial upgrading—the lower this proportion, the higher the degree of industrial upgrading. Therefore, the impact of the policy on industrial upgrading is negative rather than positive. The corrected paragraph appears below:

“The dual-pilot policy promotes work safety governance through industrial upgrading. As shown in Table 6, column (3), the policy has a significant negative effect on industrial upgrading at the 1% significance level, demonstrating that policy-driven industrial transformation helps reduce the likelihood of accidents. By advancing smart manufacturing and automation, optimizing production processes and organizational structures, enhancing production efficiency, and minimizing manual operations, the policy provides systemic support across multiple dimensions, including safety concepts, institutional norms, and technical equipment, jointly driving improvements in work safety governance. Thus, Hypothesis H3 is supported.”

The original version of this article has been updated.

